# What are patients’ knowledge, expectation and experience of radial extracorporeal shockwave therapy for the treatment of their tendinopathies? A qualitative study

**DOI:** 10.1186/s13047-018-0254-5

**Published:** 2018-04-05

**Authors:** Raymond Leung, Nikolaos Malliaropoulos, Vasileios Korakakis, Nat Padhiar

**Affiliations:** 1 Sports and Exercise Medicine Clinic, Thessaloniki, Greece; 2 National Track & Field Centre, Sports Medicine Clinic, Thessaloniki, Greece; 3 European Sports Care, London, UK; 4 Sports Clinic, Rheumatology Department, Barts Health Trust, London, UK; 5 William Harvey Research Institute, Centre for Sports and Exercise, Barts and The London School of Medicine and Dentistry, Queen Mary, University of London, London, UK; 6 Aspetar, Orthopaedic and Sports Medicine Hospital, Doha, Qatar; 7 Hellenic Orthopaedic Manipulative Therapy Diploma, Athens, Greece

**Keywords:** Experience, Extracorporeal, Knowledge, Shockwave, Tendinopathy

## Abstract

**Background:**

Extracorporeal shockwave therapy (ESWT) is used to manage different tendinopathies and appears to be effective in some tendinopathies but not others. The reasons for this are unclear. There is evidence that patient outcomes can be influenced by a patient-centred approach. There is therefore a need to qualitatively evaluate patient experiences for a treatment like ESWT where uncertainties exist. The aim of this study was to understand patients’ overall perspective of ESWT to manage their tendinopathy.

**Methods:**

A qualitative semi-structured face-to-face interview study design was used and the data was analysed thematically using ‘Framework Analysis’.

**Results:**

Eleven participants that have had radial ESWT (rESWT) to treat a range of tendinopathies were recruited from a private London sports clinic and interviewed in person or via Skype™. Four main themes and 16 subthemes were identified. Subthemes included previous failed treatment, clinician factors, mechanisms of ESWT, positive aspects, negative aspects, responsibility over own health and perceived outcomes.

**Conclusion:**

The participants understood the procedural aspects of rESWT, but were largely unaware of its mechanism of action and whether it was found to be effective for their condition or not. The participants felt that self-management measures were equally or more important than rESWT to help treat their tendinopathies. Recommendations would be for rESWT providers to offer patients written information, maintain continuity of care, address patients’ expectations, feedback on progress, and encourage self-management measures such as activity modification.

**Electronic supplementary material:**

The online version of this article (10.1186/s13047-018-0254-5) contains supplementary material, which is available to authorized users.

## Background

The commonest cause of tendon injury is from tissue overload [1] in sports [2]. The most susceptible tendons include the Achilles, patellar, posterior tibialis and rotator cuff [3]. Approximately 80% of overuse tendinopathies respond to conservative measures within 3–6 months [4]. Eccentric exercises have shown to be an effective treatment [4, 5], but there is limited evidence to support this over other conservative therapies such as massages [6].

Extracorporeal shockwave therapy (ESWT) is a safe non-invasive procedure [7] in which a device delivers acoustic energy (shockwaves) through the skin surface onto the affected area. Focused shockwaves are typically generated by electromagnetic or piezoelectric techniques [8]. Radial shockwave (rESWT) is non-focused and generated by a ballistic source [9]. National Institute for Health and Clinical Excellence (NICE) recommends ESWT should only be used with arrangements for clinical governance or research in refractory cases of lateral epicondylitis (LE), Achilles tendinopathy (AT) and plantar fasciitis (PF) [10–12].

ESWT may promote the release of angiogenetic growth and proliferating factors (e.g. vascular endothelial growth factor and endothelial nitric oxide synthase) that induces neovascularisation for tissue regeneration [8]. Furthermore, ESWT may down-regulate inflammatory mediators and directly suppress nociceptors by hyperstimulation analgesia [13].

Studies on ESWT in tendinopathy conditions (TC) have been quantitative with the use of validated outcome measures such as the visual analogue scale for pain [9], ‘Patient-Rated Tennis Elbow Evaluation’ for LE [14], and ‘Roles and Maudsley’ score for both pain and function [15]. ESWT only demonstrates favourable outcomes in certain TC such as AT [16] and calcific rotator cuff tendinopathy [9]. However, it has no significant benefit in other conditions such as LE [17] and its mechanism of action remains unknown. It is therefore important for patients to understand this information and make an informed decision about treatment.

A questionnaire study involving 865 participants at three different primary care practices found that patients wanted a patient-centred approach to their consultations [18]. The main domains were to explore patients’ experience of disease through their ideas about the problem and expectations for the visit. Another study found that patient centred score was positively associated with patient satisfaction and beneficial health outcomes [19]. These studies indicate a need to understand patients’ knowledge and expectations for any treatment they receive.

A review of randomised controlled trials (RCT) and analytical studies of physician-patient communication demonstrated an association between effective communication and improved physiological health [20]. Furthermore, a systematic review of case studies identified an association between positive patient experiences and positive clinical outcomes ranging from physical symptoms to adherence to treatment [21].

There is therefore a need to evaluate patient experiences for a treatment like rESWT whereby its mechanism and effectiveness is not fully understood. Currently, there are no qualitative studies on rESWT in TC to explore patients’ knowledge and experiences of this treatment, which could potentially identify ways to improve patient care in clinical practice.

## Method

### Study aims

1. Determine patients’ understanding of rESWT and elicit their expectations of treatment
2. Explore patients’ experiences and views of rESWT
3. Identify ways to enhance the quality of patient care and experience of rESWT.

### Design

A qualitative study design with semi-structured face-to-face interviews was used to allow participants to express their views and experiences in rich detail, and maintain some structure to address the study’s objectives [22].

### Ethical approval

Ethical approval was obtained from Queen Mary University of London Research Ethics Committee with a letter of agreement from European Sports Care (ESC) at Harley Street in London. The study complied with ethical protocols and consent was obtained from each participant.

### Participants

A purposive sampling was used to recruit participants with tendinopathies that have had rESWT (Table 1). Participants were recruited from European Sports Care (ESC), a private sports clinic at Harley Street in London. A diverse range of tendinopathies, age groups and genders were targeted for a representative sample.

**Table 1:** Inclusion and exclusion criteria

Inclusion criteria	Exclusion criteria
Age ≥ 18	The participant must be a patient of the healthcare professional providing the overall rESWT treatment and have no other affiliation
Able to give informed consent	Had or plans to have rESWT for a bone or joint specific condition such as adhesive capsulitis
Able to adequately understand written and verbal English	Had or plans to have rESWT for a muscle specific condition such as myofascial pain syndrome and myositis ossificans
Had or plans to have rESWT for a tendinopathy condition inclusive of plantar fasciitis, iliotibial band syndrome and greater trochanteric pain syndrome	

### Data collection

Participants that met the criteria were identified by the lead health care professionals (HCP) at ESC. These participants were contacted by email, post or text message with details about the study. Participation was entirely voluntary with no coercion.

The semi-structured interviews were conducted face-to-face in a mutually convenient location or via Skype™ online video-calls. A topic guide (Table 2) was developed with reference to the research question and from reviewing other qualitative studies on patient experiences [23, 24]. The topic guide ensured consistency in key areas of questioning (internal validity) whilst maintaining flexibility in participants’ responses.

**Table 2:** Topic guide

Introduction
Introduction of interviewer and overview of the study Discussion on consent, confidentiality and the interview procedure Check if interviewee has any questions and happy to proceed
Background factors
The type of tendinopathy and length of time interviewees have had it for The type of physical activities interviewees perform In what way did the condition present as a problem for the interviewees? What treatments have they tried so far or prior to rESWT if applicable? If other treatments helped, ask in what way did it help?
Preconceptions and expectations of rESWT
How did interviewees first hear about rESWT? If applicable, cover any background reading done What did interviewees understand about how rESWT works? What did interviewees understand about the procedure itself? Cover any side-effects What did interviewees expect would be the outcome after rESWT? Short and long term outcomes Discuss what interviewees personally would consider a successful outcome
Experiences of rESWT (if applicable)
What were interviewees told about rESWT by the healthcare professional? If this has already been covered from previous questions then omit If applicable, ask how interviewees feel this changed what they initially understood and expected from the procedure What were interviewees’ first experiences of rESWT like? Cover number of shocks, interactions with the professional performing the procedure, positive/negative effects and experiences Did interviewees feel their experience of rESWT was what they expected? If so, why? If not, why not? Cover anything that was done well and anything that could have been done better What are interviewees’ current views about rESWT? Relevant or not relevant to the interviewees’ tendinopathy condition Cover interviewees’ perceived outcome from the procedure (short and long term)
Close interview
Summarise discussion Reiterate confidentiality Invite any questions Thank interviewees

All the interviews were conducted by the main author and continued until no new themes emerged (data saturation). Both these aspects enhanced internal validation of the study. Fieldwork notes were taken and the audio of all the interviews were recorded, fully anonymised and identified by code only.

### Data analysis

All the interviews’ audio data were transcribed verbatim and ‘Framework Analysis’ [22] was used to analyse the data thematically by:

1. Familiarisation of the transcripts and fieldwork notes.
2. Identifying important themes and divide these into subthemes via ideas articulated within the transcripts to develop a thematic framework.
3. Indexing the data by coding the transcripts using NVivo 11 software (QSR International Ltd), then adapting the framework in light of any gaps.
4. Charting (summarising) the data within the analytical framework into manageable segments.
5. Mapping and interpreting the themes and subthemes to clarify concepts, establish links and understand the data in relation to the study’s objectives.

Respondent validation was conducted via email with 5/11 voluntary participants to ensure their data was correctly interpreted and the findings were complete.

## Results

Eleven participants (8 males and 3 females) took part in the study with a mean age of 40.54 (range 30–54). Ten participants had 9 different lower limb tendinopathies and 1 participant had 2 different upper limb tendinopathies. Three participants were primarily runners and the remainder played a mixture of sports. Seven participants completed their rESWT course and 4 participants were partially through their rESWT course. Mean total number of weekly rESWT sessions was 6.33 (range 2–10). Table 3 summarises the sample characteristics.

**Table 3:** Sample characteristics

Px	Age	Gender	Ethnicity	Diagnosis	Duration of symptoms	rESWT treatment	#* rESWT sessions	Physical activities	Pre-rESWT Tx tried
01	43	Male	Caucasian British	Left proximal hamstring td	4–5 months	Complete	8	Running (4–5 times / week at about 20 miles)	Physiotx
02	54	Female	Caucasian British	Left insertional Achilles td	11 months	Complete	10	Gym - cross trainer, treadmill	Physiotx HVI
03	51	Female	Caucasian British	Right plantar fasciitis	20 years (3–4 years persistent)	Complete	10	Horse-riding	Physiotx Acp Strassburg sock CSI
04	44	Male	Caucasian British	Right patellar td	23 months	Complete	5	Running (6 times/week - 40 miles)	Physiotx
05	35	Male	Caucasian British	Left ITB syndrome	3–4 months	Complete	8	Running (30 miles/week)	Physiotx
06	30	Female	Caucasian British	Bilateral FHL and FDL td	7 years	Complete	10	Dancing (ballet, contemporary)	Physiox Dry-need. Acp HVI Ostenil/CSI
07	45	Male	Caucasian British	Bilateral plantar fasciitis	3 years	Complete	3	Martial arts Cycling Pilates	Physiotx Gastroc release.
08	36	Male	Caucasian Spanish	Right insertional adductor longus td	2 months	Incomp.	6	Cycling Running Football Skiing	Oral NSAIDs
09	32	Male	Caucasian British	Left proximal hamstring td	7–8 years	Incomp.	5	Football (semi-professional) Cycling Rowing	Physiotx DTM Acp CSI
10	33	Male	Caucasian Greek	Right insertional infraspinatus td and Right distal MTJ biceps td	3.5–4 years	Incomp.	4 on shoulder 2 on bicep	Kickboxing	Physiotx Oral NSAIDs Acp CSI
11	43	Male	Caucasian British	Bilateral mid-portion Achilles td	1 year	Incomp.	5	Running (20 km/week) Football (five-a-side twice a week) Pilates	Self-taught stretches

*Px* participant/patient, #* number of weekly, *tx* treatment, *Incomp* incomplete, *td* tendinopathy, *ITB* iliotibial band, *FHL* flexor hallucis longus tendinopathy, *FDL* flexor digitorum longus, *MTJ* musculotendinous junction, *Physiotx* physiotherapy, *HVI* high volume injections, *Acp* acunpuncture, *CSI* corticosteroid injections, *Dry need.* Dry needling, *Gastroc*. gastrocnemius, *NSAIDs* non-steroidal anti-inflammatory drugs, *DTM* deep tissue massage

The interviews ranged from 10 min and 27 s to 28 min and 16 s in length with a mean average duration of 20 min. 4 main themes and 16 subthemes were identified in the final analytical framework (Table 4). A more detailed data charting within the analytical framework is provided in Additional file 1: Table S1.

**Table 4:** Summary of the final analytical framework

Main themes and subthemes	Analysis	Illustrative quotes
1. Choice of rESWT		
1.1 Persistent symptoms	Most had chronic symptoms that were up to many years before rESWT was tried. A mixture of emotions was expressed from some subjects with persistent symptoms – anger, hopelessness, and desperation.	“*I was so desperate for anything to help my plantar fasciitis that I was prepared to try anything, I didn't actually care what the procedure involved*” (P7). *“I was keen to investigate something that I hadn't tried before, purely because I almost ran out of options” *(P9).
1.2 Previous failed treatments	Physiotherapy, home exercises, massages, acupuncture, oral medication, injection therapy, surgery. Combination of these treatments. Misdiagnosis and delayed diagnosis was also mentioned.	“*I'd had quite a few invasive procedures, you know all the injections and things, umm and I'd also had like compartment pressure testing and everything, so to me, I was like, kind of, quite reasonably happy to go and have the ESWT*” (P6).
1.3 Non-invasive treatment	rESWT generally acceptable and preferred over more invasive therapies such as injections and surgery.	*“it seems very non invasive and if that was going to work then I'd be very happy with that”* (P2)*.* *“I think surgery was discussed, but they said they'd like to try shockwave therapy first because it was less intrusive and less risky than surgery”* (P9)*.*
1.4 Clinician factors	Reported success rates with rESWT, experience with athletes, research active, non-surgical approach.	“*He [has a] background in sports medicine and he treated not only like, every day people”* (P5). “*special to this sports medicine rather than, you know, being a general orthopod.......... I can have very much more precise conversations about training plans*” (P7).
2. Preconceptions of rESWT		
2.1 Initial knowledge about rESWT	Most had no prior knowledge about rESWT until it was introduced by a clinician and others had done their own research primarily online.	“*I don't think there was maybe that much sort of like stuff on the internet about it really*” (P4)*.* “*I had a look on pubmed, and clinical evidence for it, which seemed to be equivocal”* (P7).
2.2 Understanding of the mechanisms of ESWT	Inflammatory response, breakdown of calcium deposits, breakdown of “scar” tissue, remodelling, angiogenesis, and stimulate blood flow to the injured area were mentioned by some.	*“shockwave was going to disperse any calcium deposits I had, and also sort of agitate the tendon so it heals better”* (P2). *“stimulates more blood flow into a particular area.......... focuses more blood flow, which should improve healing” *(P9).
2.3 Initial outcome expectations	Complete resolution of symptoms, pain free, short-term gains, and return to previous sporting activity were mentioned. Those with more longstanding symptoms were less optimistic.	*“I don't think I was expecting very much to be honest because I tried so many different things. So I wasn't really expecting that much”* (P3). *“I was confident that it was going to address the problem”* (P4).
3. Experience of rESWT		
3.1 Initial discussion about rESWT with the healthcare professional	Description about the rESWT procedure itself, the protocol of requiring a number of weekly sessions, and, in some cases, potential mechanisms, side-effects, post-procedural practical advice were discussed.	*“avoid long walks immediately following the treatment for the next day”* (P3). *“there's firing [of] this ball bearing at high frequency into the damaged area, and the whole idea about it, you're damaging the tendon, to basically get it to repair itself” *(P4)*.*
3.2 First experiences of rESWT	Generally considered an unfamiliar sensation.The initial experience of rESWT ranged from being comfortable to being significantly painful.	“*it was really quite pleasant, and particularly after the treatment, it felt, my foot felt great”* (P3). *“really, really painful, really uncomfortable”* (P4)*.* *“I'd describe it as less painful than deep tissue massage” *(P9)*.*
3.3 Subsequent experiences of rESWT	Increase intensity with subsequent sessions did not always reflect a more painful experience and tolerance maybe a factor as well as noticeable improvements in symptoms.	*“I was use to quite sharp pain shooting through the tendon. The ESWT to me seemed kind of like, ok, in comparison because it was more like a kind of ache or pressure”* (P6). *“you know if I can't walk to the shops or I can't take my children for a walk ..........frankly, two minutes of pain with a thing hammering at my foot was completely irrelevant” *(P7).
3.4 Experiences of rESWT against expectations	Uncertainty as to what to expect. Comparisons of rESWT experience with other previous treatments and attitudinal factors had an influence on expectations.	*“I wasn't thinking it wasn't going to work at any one point.......I felt something was being done”* (P2). *“it's not as frightening as other stuff because it's non-invasive, so it's not like someone's saying to you, right, I'm going to chop you open and I'm going to take this out, and I'm going to stitch that together”* (P5)*.* *“I expected it to be more painful or kind of hurt in a different way if that makes sense. I think also if you've had like lots of invasive treatments, you do expect it to kind of hurt in a kind of way like an injection would”* (P6)*.*
3.5 Aspects that were done well (Positive aspects)	Healthcare professional periodically checking to see if the patient was tolerating the rESWT, being able to feedback on progress, provision of pre-planned exercises, and other treatment options. A friendly, knowledgeable and sensitive medical approach to the problem.	*“I felt like I was getting answers to my questions and obviously, I had a good idea of how things were progressing”* (P4). *“when you go to the physio, it was always really annoying that you had to wait 2 weeks before you know, you increase the exercises, whereas he [sports consultant] kind of pre-planned for, this is the easy, medium, hard version”* (P6). *“he personalised the treatment, so the number of sessions and I expect also the impact of shockwave” *(P7).
3.6 Aspects that were not done well (Negative aspects)	Procedure protocol, success rates, outcome timeframes, side-effects and activity modification measures were not always adequately covered for some patients. Unable to understand or address the root cause of the tendinopathy.	*“throughout the treatment it got tougher, and I don't remember that being explained to me”* (P3). *“what about the cause?................ my only one little bit of frustration was that maybe we should have addressed this VMO stuff earlier”* (P4). *“I wasn't allowed to dance whilst doing it I suppose. I suppose actually that's probably the only thing that I wasn't aware of until the first treatment............I had to suddenly make plans”* (P6).
4. Current views of rESWT		
4.1 Personal views and level of improvement	Overall rESWT was found to have a positive effect on the different tendinopathy conditions to a varying degree but had not cured the problem for most. Some considered other factors (e.g. rest, activity modification, appropriate exercises) to have improved symptoms rather than rESWT alone.	*“I run the Brighton half marathon in an hour and 35.......without pain and probably quicker..........I'm convinced it's all of 80% shockwave and then with the strengthening and that, made it 100[%]”* (P5)*.* *“I had the second course [of ESWT] which improved it probably about 90% better, it didn't quite clear it, but I had a third course which cleared it on the right foot completely, and almost cleared it on the left foot”* (P7). *“I guess the bit I don't know, is whether I would be in the same place having not had the procedure because of natural healing or not?”* (P8)*.*
4.2 Responsibility over own health	Awareness that physical activity modification and exercises were important alongside rESWT.	*“I have now lost nearly 3 stone, so I'm thinking if I could lose more weight, I might be able to put off any other further problems”* (P2). *“He got me to get like a trampette and to get like a wobble board and do quite dance specific, injury specific exercises”* (P6).
4.3 Perceived outcomes	Remain asymptomatic and return to baseline health status in a minority. For those that had not completed their course of rESWT they were not confident that the condition will be cured completely.	*“I can walk at work without being in pain. So, to me, that's massive, so you know, I'm doing all of the same on-calls, the same duties, walking around buildings and it doesn't hurt anymore, so to me that's a massive positive even though I know, at the minute, I can't dance for hours on end”* (P6). *“these issues I'd say they are quite resistant so far, for quite a long time, so if they completely disappear, that would be great, but I'm not sure if this is going to happen”* (P10)*.*

### Choice of rESWT

Majority of participants had chronic symptoms with previous failed treatments that influenced them to consider rESWT, which was unheard-of by many. There were a mixture of anger, hopelessness and desperation to manage their conditions. A minority had ineffective surgery or were previously misdiagnosed.

The non-invasive nature of rESWT and certain clinician factors were important for some participants’ decision-making. Clinician factors included specialising in sports medicine, knowledgeable about musculoskeletal rehabilitation and being research active with rESWT. There were verbal discussions from the clinician about favourable success rates of rESWT in TC, but participants were not necessarily presented with data to backup these claims.

### Preconceptions of rESWT

Participants mainly used the internet to learn more about ESWT. Some focussed on procedural aspects of rESWT, and some read journal articles with conflicting results on ESWT effectiveness. Others did no background reading for reasons such as lack of time, no interest, and concerns about the reliability of online information. Most participants had no understanding of the proposed mechanisms of ESWT on TC. Two participants (P6 and P9) had some understanding, and two participants (P2 and P11) referenced the ESWT effects on calcium deposits. Pre-treatment outcome expectations of rESWT ranged from symptom control to complete resolution. Those that had more longstanding symptoms were less optimistic.

### Experiences of rESWT

The HCP gave participants descriptions about the rESWT procedure and its weekly protocol. Most were informed about common side-effects (e.g. pain and swelling) but only a minority were told about major complications such as tendon rupture. Discussions regarding contraindications were not reported. Practical post-procedural advice (e.g. avoid long walks after rESWT for PF) were discussed.

First experiences of rESWT were generally considered an unfamiliar sensation. Some participants found it therapeutic whilst others found it excruciatingly painful. Side-effects experienced beyond pain were rare. Increased rESWT energy did not always correlate with a more painful experience. Experiences of rESWT against expectations were matched for some participants but others were unsure what to expect.

Positive aspects of treatment included general rapport with the HCP, the HCP’s periodic assessment of participants’ rESWT tolerance, treatment personalisation, and weekly progress monitoring. Those that had pre-planned exercises in their overall management found this useful. Negative aspects of treatment for a minority of individual participants, included inadequate information provided about the procedure protocol, success rates, outcome time-frames, side-effects and activity modification requirements. One participant (P8) reported disparities in rESWT techniques between two different HCPs, and two participants (P1 and P4) thought that the underlying cause of their TC was not addressed.

### Current views of rESWT

Overall rESWT had a positive effect for all the participants’ TC to a varying degree. Most had gains to continue their daily activities but not their previous physical activity level. Those that completed the rESWT course reported an improvement between 50 and 100% (mean 81.4%). Those that had not yet completed the rESWT course did not perceive that their TC would be cured. Half that had not yet completed their treatment course quoted 30% and 45% improvement, whilst the remainder wanted to reserve judgement until they finish the course.

Many participants conveyed the importance of implementing lifestyle changes, modifying physical activities, and maintaining specific exercises. Some felt that these factors were more significant to their level of improvement than rESWT alone.

## Discussion

The study was the first to evaluate patients’ knowledge, expectations and experiences of rESWT via interviews. Four main themes about rESWT were derived from the study: choice of treatment, preconceptions, overall experiences and current views on the treatment. Knowledge about rESWT involved understanding the procedure and awareness of its non-invasive nature. The decision to have rESWT was primarily influenced by the HCP, and the expectations of treatment were affected by the chronicity of patients’ symptoms. Overall positive patient experiences related to empathic care delivered by the HCPs, and negative experiences involved inadequate information provided about aftercare.

rESWT was an acceptable treatment due to its non-invasive nature. Chronic symptoms and other previous failed treatments influenced participants’ decision to have rESWT. These factors should be addressed by HCPs who should also recognise that their own expertise and rapport with patients plays a role in shared decision-making. These findings were consistent with a study that found patients’ involvement in decision-making relies on an effective relationship with the HCP [25].

Participants wanted to know about the success rates of rESWT for their TC, and whilst this was sometimes discussed by the HCP, it was unclear whether success rates were taken from audit data within the private clinic or from existing published trials. Systematic reviews have demonstrated effective outcomes for ESWT on PF and AT [9], and limited evidence for patellar tendinopathy [16]. A small RCT has found rESWT to be effective in 85% of professional athletes with chronic proximal hamstring tendinopathy [26]. There have been no published human studies on ESWT for biceps tendinopathy, flexor hallucis longus tendinopathy and flexor digitorum longus tendinopathy. It remains to be known whether provision of this research data to the participants would have made any impact on their decision to have rESWT.

Internet resources were primarily used by most participants to review ESWT effectiveness, but there were concerns over the credibility of the sources found online. Therefore, concise patient information leaflets on rESWT should be widely available from reliable healthcare websites (e.g. NHS Direct). Those uninterested in medical literature should still be given this information to encourage involvement in their care [18]. The proposed mechanisms of ESWT to manage tendinopathies were not well understood by participants either because they lacked understanding of medical terminologies (e.g. scar tissue) or they were not provided with this information. These aspects of treatment should be discussed with patients. Majority expected rESWT to provide symptom control as a minimum, but more chronic symptomatic participants displayed less optimism and it was important to address this early because positive expectations in those with chronic pain are linked with superior treatment gains [27]. Side-effects beyond pain were rarely reported and these findings were consistent with a systematic review that found no serious adverse events with ESWT [28]. However, contraindications of ESWT [29] must always be checked for.

Experiences of rESWT were different between participants even with the same condition. Positive experiences were attributed to willingness to complete the treatment course, and noticeable improvement in symptoms with subsequent sessions. The increase pain tolerance with subsequent rESWT sessions corresponds with hyper-stimulation analgesia pathways [13]. Optimistic attitudes towards treatment and reassurance of its non-invasive nature, positively influenced the experience and expectations of treatment in some participants.

An overall provision of empathic care and continuity of care were the most positive aspects of management. Continuity of care has been linked with patient satisfaction [30]. All these participants had a personalised number of shockwave sessions tailored towards their progress which they themselves favoured. Other rESWT providers follow a standardised protocol [31]. A review has recommended that ESWT protocols should be individualised in order to adapt to different stages of a given tendon pathology that will respond to ESWT differently [32]. A retrospective study [33] used an individually adapted rESWT protocol for plantar fasciopathy and found promising results.

Negative experiences of management were related to areas that were not fully addressed. All efforts should therefore be made to allow patients to feedback and ask questions. Two participants wanted to understand the causation of their tendinopathy and perhaps would have benefited from additional physiotherapy and biomechanical input.

All participants that completed their rESWT course within this small cohort subjectively felt rESWT was effective especially towards resuming their daily activities. Many participants felt that appropriate exercises and lifestyle measures were important to their recovery, and rESWT alone was inadequate. These findings correlate with evidence that tendinopathy treatment mainly requires stimulation of the tendon to improve its capacity through structured loading and monitoring of tissue response to load [5, 34, 35]. Therefore, rather than identify individual modalities to treat tendinopathies [36], a multidisciplinary approach with emphasis on patient self-management should be incorporated.

### Limitations

The participants were only recruited from one private clinic under mainly one clinician, so therefore the findings are subjected to selection bias and are not transferable in other settings, locations or patient groups (e.g. ESWT through the National Health Service). The sample frame consisted of a mixture of gender, age groups and TC, but a lack of ethnic diversity further impacted on how representative the sample was.

Although data saturation was achieved, the overall sample was small and homogenous. Recruitment from a single clinic potentially limited the number of emerging themes. Recall bias was an issue for those that had rESWT over months or years prior to the interview and, moreover, the requirement for voluntary input from respondents subjected the study to recruitment bias.

Respondent validation clarified the accurate interpretation of the data, but triangulation of the data with different analysis and involvement of other investigators was not performed. The study was unique, but this meant that findings were not comparable to other similar studies.

## Conclusion

In this study participants were largely unaware of how rESWT works and whether it was effective for their condition, so these factors need to be discussed to ensure patients can make an informed decision. A further recommendation would be for rESWT clinics to offer patient information leaflets and for reputable health care websites to develop patient resources on rESWT. Participants had their own defined expectations from rESWT that needs to be addressed early. The study also highlighted patient awareness of the importance of lifestyle measures, physical activity modification and appropriate exercise programmes. These behavioural changes and self-management measures should always be encouraged. Demonstrating good standards of empathic care, providing continuity of care, encouragement of patient feedback should be promoted in all ESWT clinics. Further qualitative studies should recruit a diverse population of patients from both private and public sectors, and examine the views of HCPs providing rESWT for patients.

## Additional file

Additional file 1: **Table S1.** Data charting within the analytical framework. (DOCX 28 kb)

## Abbreviations

AT: Achilles tendinopathy
ESC: European Sports Care
ESWT: Extracorporeal shockwave therapy
HCP: Health care professional
LE: Lateral epicondylitis
NICE: National Institute for Health and Clinical Excellence
PF: Plantar fasciitis
RCT: Randomised controlled trials
rESWT: Radial extracorporeal shockwave therapy
TC: Tendinopathy conditions
