# Supplementary material for: What are patients’ knowledge, expectation and experience of radial extracorporeal shockwave therapy for the treatment of their tendinopathies? A qualitative study
Source: J Foot Ankle Res. 2018 Apr 5;11:11. doi: 10.1186/s13047-018-0254-5 (PMC5887221; doi:10.1186/s13047-018-0254-5)
Supplement: Supplementary file 1 — Table S1. Data charting within the analytical framework. (DOCX 28 kb) [file 13047_2018_254_MOESM1_ESM.docx]

**Table S1: Data charting within the analytical framework**

| **Cases** | **Chart 1: Choice of rESWT** | | | |
| --- | --- | --- | --- | --- |
|  | 1.1 Persistent symptoms | 1.2 Previous failed treatments | 1.3 Non-invasive treatment | 1.4 Clinician factors |
| Subject 1  Age: 43  Gender: Male  Ethnicity: Caucasian British  Physical activities:  running 4-5 times per week at about 20 miles  Diagnosis: left proximal hamstring tendinopathy  Initial presentation: during running at boot camp  Symptom duration: 4-5 months  Treatments prior to rESWT:  Physiotherapy  Number of weekly rESWT: 8  rESWT course complete: Yes | Not discussed whether persistent symptoms were a factor that influenced him to have rESWT | He initially went through his insurance company and saw a physiotherapist where he was given exercises and stretches to do. He also stopped running and focussed on static exercises. Despite these measures his pain and irritation persisted, so he went through the insurance company again that directed him to a sports consultant. | When initially asked whether he thought rESWT was going to be invasive after he did his own research he replies, *“I really didn’t know”*, but then recalls looking at ESWT images online and possibly knew to a certain extent it was non-invasive. However, this was not a factor that influenced his choice on having rESWT. | Not reported clinician factors to have influenced his decision to have rESWT, instead it was introduced to him as a treatment option and he accepted it. |
| Subject 2  Age: 54  Gender: Female  Ethnicity: Caucasian British  Physical activities:  Gym – cross-trainer, treadmill  Diagnosis: left insertional achilles tendinopathy  Initial presentation: gradual after a 4.5 mile walk  Symptom duration: 11 months  Treatments prior to rESWT:  Physiotherapy, high-volume injections,  Number of weekly rESWT: 10  rESWT course complete: Yes | Despite she had the symptoms for about 11 months before rESWT, the symptoms actually improved with different treatments in between but the symptoms would flare-up after her travels. | She had good success with high volume injection where the described the *“relief was instantaneous”*. She did not comment on how helpful physiotherapy was prior to rESWT but viewed it as a supportive treatment for the tendon. | rESWT was recommended to her by the sports consultant following an ultrasound evaluation of her achilles. She comments on rESWT that *“it seems very non invasive, umm and if that was going to work then I’d be very happy with that”*. | Her decision was influenced by the clinician’s reported success rates and confidence. She can recall being told only a small number of patients that had rESWT was unsuccessful “*there was one, one or two people at the most that he hasn’t actually helped. So I was very hopeful going into the treatment that it was going to work*”. |
| Subject 3  Age: 51  Gender: Female  Ethnicity: Caucasian British  Physical activities:  Horse-riding  Diagnosis: right plantar fasciitis  Initial presentation: gradual with activities of daily living  Symptom duration: 20 years but more persistent over 3-4 years  Treatments prior to rESWT:  Physiotherapy, acupuncture, Strassburg sock, steroid injections  Number of weekly rESWT: 10  rESWT course complete: Yes | Not discussed whether persistent symptoms were a factor that influenced her to have rESWT. | Had a range of therapies – physiotherapy, acupuncture, home remedies (ice bottles underneath feet), Strassburg sock, steroid injection. It was clear none of these were effective as she appeared annoyed when discussing these therapies and she discusses her expectations of rESWT (see 2.4). | Not discussed whether the non-invasive nature of rESWT was a factor that influenced her decision to have rESWT although she was aware it was not going to be invasive. | Not reported clinician factors to have influenced her decision to have rESWT. She had already done some research about ESWT beforehand. |
| Subject 4  Age: 44  Gender: Male  Ethnicity: Caucasian British  Physical activities:  running 6 times per week at 40 miles  Diagnosis: right patellar tendinopathy  Initial presentation: progressive pain after running  Symptom duration: 23 months  Treatments prior to rESWT:  Physiotherapy  Number of weekly rESWT: 5  rESWT course complete: Yes | He emphasised that despite not running for a month it didn’t make any difference to him. So he wanted to try treatment even if it meant he had to pay for it himself, but on the assumption that it would work as he says, *“happily pay £500 to be able to umm run pain free”* | He was originally misdiagnosed by the physiotherapist with medial tibial stress syndrome and expressed disappointment with this at which point he was then introduced to rESWT. | Not discussed whether the non-invasive nature of rESWT was a factor that influenced his decision to have rESWT | He actually saw two clinicians. The original clinician he saw was a runner as well so he felt that a clinician *“being able to understand runners”* was relevant to him understanding the rESWT. For the second clinician who was the sports consultant, he expressed that the clinician’s *“experience with athletes”* was also an important factor in his decision to have rESWT |
| Subject 5  Age: 35  Gender: Male  Ethnicity: Caucasian British  Physical activities:  running 30 miles per week  Diagnosis: left iliotibial band syndrome  Initial presentation: sudden sharp pain in left knee  Symptom duration: 3-4 months  Treatments prior to rESWT:  Physiotherapy  Number of weekly rESWT: 8  rESWT course complete: Yes | Not discussed whether persistent symptoms were a factor that influenced him to have rESWT | He paid for private physiotherapy initially that did not help, so he then went to his GP to be referred privately using his company’s medical insurance, and saw a sports consultant. | He was keen to avoid injections as this was an intervention that he was aware of and he was informed by the sports consultant that it could weaken the tendon even more. His remarks on injections were, *“it's invasive as well and no one likes getting jabbed with needles really”* | He was provided with some information by the sports consultant that rESWT has been used to treat professional athletes that seemed to instil some confidence in him to have rESWT as he says of the sports consultant, “*He (has a) background in sports medicine and he treated not only like, every day people*” |
| Subject 6  Age: 30  Gender: Female  Ethnicity: Caucasian British  Physical activities:  dancing, ballet  Diagnosis: bilateral flexor hallucis longus and flexor digitorum longus tendinopathy  Initial presentation: imbalance whilst doing pointe work  Symptom duration: 7 years  Treatments prior to rESWT:  Physiotherapy, dry-needling, acupuncture, high volume injections, Ostenil injections, steroid injections  Number of weekly rESWT: 10  rESWT course complete: Yes | She has had her tendinopathies since 2007 and there was a sense of hopelessness when she was reflecting on having it for such a long period of time. She had insight that it was caused and exacerbated by her dancing and ballet. | She was initially diagnosed with chronic compartment and had a fasciotomy first, to which she laughs as she reflects on all the various treatments she has been through. Only after the fasciotomy did the clinicians *“then realised that actually there was underlying tendinopathy the whole time!”* as she expresses some frustration. Whilst this was not a misdiagnosis, it was a late diagnosis.  She had physiotherapy, high volume injections, ostenil injections, dry-needling, steroid injections and acupuncture. She states that “*I’d had quite a few invasive procedures, you know all the injections and things, umm and I’d also had like compartment pressure testing and everything, so to me, I was like, kind of, quite reasonably happy to go and have the ESWT*”. | She had done some prior research on ESWT (see 2.1) so was aware it was a non-invasive procedure. She felt that as she had so many invasive procedures already, she was happy go for this non-invasive treatment – “*to be honest, I’d had quite a few invasive procedures, you know all the injections and things, umm and I’d also had like compartment pressure testing and everything, so to me, I was like, kind of, quite reasonably happy to go and have the ESWT done*”. | Not reported clinician factors to have influenced her decision to have rESWT. |
| Subject 7  Age: 45  Gender: Male  Ethnicity: Caucasian British  Physical activities:  martial arts, cycling, pilates  Diagnosis: bilateral plantar fasciitis  Initial presentation: after a routine run  Symptom duration: 3 years  Treatments prior to rESWT:  Physiotherapy, gastrocnemius release  Number of weekly rESWT: 3  rESWT course complete: Yes | He has had the symptoms for 3 years and due to its significant impact on his physical activities there was a sense of urgency for help as he says in a slightly humorous manner, “*I was so desperate for anything to help my plantar fasciitis that, umm, well, I was prepared to try anything haha, I didn’t actually care what the procedure involved*”. He also draws on other people’s experiences with plantar fasciitis, *“I think, speak to many people with plantar fasciitis who had it for a long time, uhh, actually [they] will do almost anything to get rid of it”*. | He has had *“extensive physiotherapy”* prior to rESWT and has also had gastrocnemius release surgery, which he reflects on regretting that decision, *“I had surgery on my legs to release the calfs, to lengthen them and umm, that, that made no difference to my plantar fasciitis”*.  He was willing to try anything that could potentially help his symptoms, *“having had surgery on both legs, haha, with all of that involved, time off work, crutch, you know, two crutches, feet up, both feet up for a long time, I thought after that, really, it couldn’t be uhh, as bad as that”*. | He did internet research (see 2.1) and thought that rESWT *“didn’t seem to be that invasive and uhh there didn’t seem to be any negatives, uhh, negative effects for it, so I thought I’d give it a try”*.  He compares the options of rESWT or corticosteroid injections, and has sought clinical advice from people he knows within the medical field, *“They said, given a choice between umm, corticosteroid injection and shockwave, they would definitely go for shockwave, so, that was that. I was convinced”*. | Clinician factors were another important aspect for his decision to have rESWT. As he says about the sports consultant, *“someone who is research active in the treatment and that’s hugely confidence inspiring”*.  He also comments on the approach of a medical doctor over a surgeon, “*he’s special to this sports medicine rather than, you know, general, being a general orthopod”* and referring the sports consultant, “*I can have very much more precise conversations about training plans*”. These comments may partially be influenced by the failed surgical treatment that he had. |
| Subject 8  Age: 36  Gender: Male  Ethnicity: Caucasian Spanish  Physical activities:  cycling, running, football, skiing  Diagnosis: right adductor longus strain  Initial presentation: rip sensation on back swing of right leg after kicking a football  Symptom duration: 2 months  Treatments prior to rESWT:  Oral NSAIDs  Number of weekly rESWT: 6  rESWT course complete: No | As his injury was relatively acute and he sought help early, persistent symptoms were not a predominant factor that influenced his decision to have rESWT. | He initially treated the injury with RICE (rest, ice, compression and elevation), had some oral anti-inflammatories, but had no subsequent treatments prior to shockwave. | He did assume rESWT was non-invasive as he makes comparisons with surgery, *“I would have imagined that having surgery would have meant a longer period just because of recovery from a surgery as well, so I was happy that that wouldn’t be the case”*. However, this was not really an influencing decision. | He was offered rESWT as an initial treatment for his adductor strain. He went for the treatment “*because the doctor recommended it, I was happy to do it*”. Perhaps there was a subtle doctor-centred approach to this consult but he himself did not express any particular interest in evaluating or researching other options. |
| Subject 9  Age: 32  Gender: Male  Ethnicity: Caucasian British  Physical activities: football (semi-professional), cycling, rowing  Diagnosis: left proximal hamstring tendinopathy  Initial presentation: sensation of being kicked in the left leg during football  Symptom duration: 7-8 years  Treatments prior to rESWT:  Physiotherapy, deep tissue massage, acupuncture, steroid injections  Number of weekly rESWT: 5  rESWT course complete: No | He expresses desperation to try any treatment that will help with his chronic symptoms, *“I was keen to investigate something that I hadn’t tried before, purely because I almost ran out of options”*. | He tried acupuncture, steroid injections and had various rehabilitation programmes that were all ineffective. Deep tissue massage was *“quite effective”* for him. | He felt that surgery was the only alternative option prior to rESWT after his other failed therapies. He made comparisons of rESWT with surgery, *“I think, surgery was discussed, umm but they said they’d like to try shockwave therapy first because it was less intrusive and less risky than surgery”*, and *“often meant that patients didn’t have to then undergo surgery, which can obviously cause complications”*. | Not reported clinician factors to have influenced his decision to have rESWT. |
| Subject 10  Age: 33  Gender: Male  Ethnicity: Caucasian Greek  Physical activities:  kickboxing  Diagnosis: right insertional infraspinatus tendinopathy and right distal musculotendinous junction biceps tendinopathy  Initial presentation: gradual with daily activities such as feeding self and showering  Symptom duration: 3.5-4 years  Treatments prior to rESWT:  Physiotherapy, oral NSAIDs, acupuncture, steroid injection  Number of weekly rESWT: 4 on infraspinatus tendon and 2 on bicep tendon  rESWT course complete: No | He expresses anger and frustration with his persistent symptoms. He says, *“I’ve been having this issue for a long time and I’m sick of it, so, guess I was willing to try anything”*. | He had oral anti-inflammatories, massages, acupuncture and steroid injections, all of which did not fully help his symptoms so this to an extent did influence his decision. | Not discussed whether the non-invasive nature of rESWT was a factor that influenced his decision to have rESWT. | Not reported clinician factors to have influenced his decision to have rESWT. |
| Subject 11  Age: 43  Gender: Male  Ethnicity: Caucasian British  Physical activities:  running (20km/week), football (five-a-side twice/week), pilates  Diagnosis: bilateral mid-portion achilles tendinopathy  Initial presentation: progressive stiffness followed by clicking  Symptom duration: 1 year  Treatments prior to rESWT:  Self-taught stretches  Number of weekly rESWT: 5  rESWT course complete: No | He had persistent symptoms for about 1 year and sought help once the pain increased but this in itself did not pre-dominantly influence his choice to have rESWT. | He did self-taught exercises and stretches but did not have any other specific therapies or guidance from healthcare professional, so failed therapies was not an influencing factor for him to have rESWT. | He recalled after his discussion with the sports consultant that he was given two treatment options – either intrusive steroid injections or non-intrusive rESWT, so he *“preferred to go for uhh on which is non-invasive”*. Perhaps from the outset he could be offered other therapies as well such as physiotherapy. | Not reported clinician factors to have influenced his decision to have rESWT. |
